# RNAseq Analysis of the *Drosophila* Response to the Entomopathogenic Nematode *Steinernema*

**DOI:** 10.1534/g3.117.041004

**Published:** 2017-04-24

**Authors:** Shruti Yadav, Sean Daugherty, Amol Carl Shetty, Ioannis Eleftherianos

**Affiliations:** * Insect Infection and Immunity Laboratory, Department of Biological Sciences, Institute for Biomedical Sciences, The George Washington University, Washington, DC 20052; † Institute for Genome Sciences, Department of Microbiology and Immunology, University of Maryland School of Medicine, Baltimore, Maryland 21201

**Keywords:** *Drosophila*, *Steinernema*, RNAseq, immunity, development

## Abstract

*Drosophila melanogaster* is an outstanding model to study the molecular and functional basis of host–pathogen interactions. Currently, our knowledge of microbial infections in *D. melanogaster* is well understood; however, the response of flies to nematode infections is still in its infancy. Here, we have used the potent parasitic nematode *Steinernema carpocapsae*, which lives in mutualism with its endosymbiotic bacteria *Xenorhabdus nematophila*, to examine the transcriptomic basis of the interaction between *D. melanogaster* and entomopathogenic nematodes. We have employed next-generation RNA sequencing (RNAseq) to investigate the transcriptomic profile of *D. melanogaster* larvae in response to infection by *S. carpocapsae* symbiotic (carrying *X. nematophila*) or axenic (lacking *X. nematophila*) nematodes. Bioinformatic analyses have identified the strong induction of genes that are associated with the peritrophic membrane and the stress response, as well as several genes that participate in developmental processes. We have also found that genes with different biological functions are enriched in *D. melanogaster* larvae responding to either symbiotic or axenic nematodes. We further show that while symbiotic nematode infection enriched certain known immune-related genes, axenic nematode infection enriched several genes associated with chitin binding, lipid metabolic functions, and neuroactive ligand receptors. In addition, we have identified genes with a potential role in nematode recognition and genes with potential antinematode activity. Findings from this study will undoubtedly set the stage for the identification of key regulators of antinematode immune mechanisms in *D. melanogaster*, as well as in other insects of socioeconomic importance.

Parasitic nematodes infect both vertebrate and invertebrate animals and cause serious diseases of socioeconomic importance (Stock 2005; Krecek and Waller 2006). The lack of good animal models has hindered the study of parasitic nematode infections in humans and agricultural pests (Hotez *et al.* 2009; Hawdon 2014). Insects have emerged as convenient models to study host responses to nematode parasitism because they share considerable homology to certain mammalian molecular factors (Loker 1994). The common fruit fly, *Drosophila melanogaster*, has been established as a supreme model organism to investigate the molecular basis of the interactions between hosts and microbes, and to identify the genetic pathways that participate in the host response to pathogenic micro-organisms due to the substantial similarities it shares with the physiological processes of vertebrate animals, including humans (Dionne and Schneider 2008; Bier and Guichard 2012; Rämet 2012).

Entomopathogenic nematodes are facultative parasites of insects, and members of the Steinernematidae family are potent pathogens of a wide range of insect species (Poinar 1972; Gaugler and Kaya 1995; Dillman *et al.* 2012). *Steinernema carpocapsae* nematodes mutualistically associate with the Gram-negative bacteria *Xenorhabdus nematophila* to invade and kill insects (Peña *et al.* 2015). These nematodes cause infections at the infective juvenile (IJ) stage, which is the developmentally-arrested third larval stage analogous to the dauer stage of the nonpathogenic nematode *Caenorhabditis elegans* (Goodrich-Blair 2007). IJs gain access to the host either by entering through natural openings or by penetrating through the insect cuticle (Arefin *et al.* 2014; Peña *et al.* 2015). Once inside the host, the IJs release their mutualistic *Xenorhabdus* bacteria, which secrete a wide range of toxins, some of which interfere with the host immune response (Clarke and Goodrich-Blair 2007). The nematodes also produce molecules that suppress or evade certain insect immune functions in order to survive and complete their life cycle in their insect host (Castillo *et al.* 2011). The nematodes reproduce using the insect cadaver as a food source and, once the resources are depleted, they reacquire the bacteria and exit as IJs in search of new prey (Goodrich-Blair 2007).

Recent studies have shown that *S. carpocapsae* is more pathogenic to *D. melanogaster* larvae compared to *Heterorhabditis bacteriophora* nematodes, which could lead to changes in the transcriptome profile of the host. Therefore, *S. carpocapsae* can be used to explore the interplay between certain aspects of the insect immune response and nematode parasitism strategies (Castillo *et al.* 2011; Peña *et al.* 2015). In a previous microarray study, the transcriptome of *D. melanogaster* larvae infected with *H. bacteriophora* nematodes was analyzed. The authors identified the participation of *tep* and *Imaginal Disc Growth Factor* (*Idgf*) genes, *Peptidoglycan Recognition Proteins* (*PGRP-LC*), and some unknown genes with putative immune function against *H. bacteriophora* nematodes (Arefin *et al.* 2014). A later study used whole-genome mRNA sequencing (RNAseq) to analyze the transcriptome of *D. melanogaster* adult flies responding to *H. bacteriophora* symbiotic or axenic nematodes or their mutualistic bacteria, *Photorhabdus luminescens*. This study revealed the participation of several different types of genes encoding lipases and heat shock proteins, as well as genes that are involved in the stress response, metabolism, and neuronal functions against these pathogens (Castillo *et al.* 2015).

Previous and recent work has demonstrated the power of using *Drosophila* for studying the molecular/genetic basis of insect immune responses against infections by entomopathogenic nematodes. Infection of *D. melanogaster* larvae with *H. bacteriophora* symbiotic nematodes results in the transcriptional activation of four antimicrobial peptide (AMP)-coding genes (Hallem *et al.* 2007). The AMP response is specific to *P. luminescens* bacteria because axenic nematodes fail to induce AMP gene transcription. We recently found that *H. bacteriophora* symbiotic and axenic nematodes induce transcription of several immune-related genes in adult flies, but that injection of *P. luminescens* bacteria alone results in lower levels of gene transcription in the fly (Castillo *et al.* 2012). Inactivation of *D. melanogaster* transglutaminase, a conserved component of clotting cascades in insects and humans, results in decreased aggregation of zymosan beads and increased sensitivity of larvae to infection by *H. bacteriophora* symbiotic nematodes (Wang *et al.* 2010). Two clotting factors (gp150 and fondue), a homolog of thioester-containing complement protein 3, a basement membrane component (glutactin), a recognition protein (GNBP-like 3), and several small peptides contribute to the immune response of *D. melanogaster* larvae against *H. bacteriophora* symbiotic nematodes (Hyrsl *et al.* 2011; Arefin *et al.* 2014). It has further been shown that *S. carpocapsae* symbiotic nematodes upregulate the expression of certain AMP genes and induce the melanization pathway in *D. melanogaster* larvae (Peña *et al.* 2015). More recently, we have found that infection with *Heterorhabditis* nematodes regulates the TGF-β pathway in *D. melanogaster* adults, and inactivation of certain TGF-β ligands modulates the survival of flies to nematode infection and the persistence of the parasites in the mutant flies (Eleftherianos *et al.* 2016).

Here, we used RNAseq analysis to investigate the transcriptomic profiles of *D. melanogaster* larvae responding to infection by *S. carpocapsae* symbiotic or axenic nematodes. Our goal was to identify the number and nature of *D. melanogaster* genes that are differentially regulated upon *S. carpocapsae* nematode infection. We have found that the *S. carpocapsae* nematode infection induces distinct types of *D. melanogaster* genes compared to infection by microbial pathogens or other nematode parasites. We have determined several genes with putative roles in the interaction between *D. melanogaster* and *S. carpocapsae* parasitic nematodes. These results set the scene for the identification of the molecular determinants of the insect immune response to entomopathogenic nematodes.

## Materials and Methods

### Fly stocks

Oregon R third instar larvae were used for the transcriptomic analysis. Flies were reared on instant *Drosophila* diet (Formula 4–24 *Drosophila* medium) supplemented with yeast (Carolina Biological Supply), maintained at 25° and a 12:12 hr light:dark photoperiodic cycle.

### Nematodes

*S. carpocapsae* entomopathogenic nematodes carrying their mutualistic bacteria *X. nematophila* were amplified in the larvae of the wax moth *Galleria mellonella* using the water trap technique (White 1927). Axenic nematodes were cultured using the Oily-agar plates protocol (Yadav *et al.* 2015). To confirm the absence of *X. nematophila* bacteria in these nematodes, a fresh pellet of IJs was collected, washed once with 1% bleach and five times with sterile distilled water, homogenized, and the lysate was spread on LB-agar plates. Absence of bacterial growth after 24–48 hr confirmed the axenicity status of *S. carpocapsae* nematodes. Nematodes with or without *X. nematophila* bacteria were used 1–3 wk after collection and nematode density was estimated in 10 µl of suspension.

### Infection assay

Microtiter 96-well plates were used for carrying out nematode infections. The plates were prepared by adding 100 µl of 1.25% agarose to each well. Sterile distilled water (10 µl) containing 100 nematodes was pipetted into the wells and an individual larva was transferred to each well. The plate was covered with a Masterclear real-time PCR film (Eppendorf) and holes were pierced for ventilation. Treatment with sterile water served as control.

### RNA isolation

Four larvae per treatment were collected at 6 and 24 hr postinfection. Total RNA was extracted using the PrepEase RNA spin kit (Affymetrix) following the manufacturer’s instructions. Total RNA was eluted in 40 µl of nuclease-free water and RNA concentration was measured using a Nanodrop (Thermo Scientific). RNA integrity and quality were estimated using a Bioanalyzer (Agilent Technologies).

### Library preparation and RNAseq

Separate libraries for the three experimental conditions (larvae infected with *S. carpocapsae* symbiotic or axenic, as well as uninfected water controls) belonging to three independent experiments were prepared with the TruSeq RNA Sample Prep kit (Illumina, San Diego, CA) according to the manufacturer’s protocol. Adapters containing seven nucleotide indexes were ligated to the double-stranded complementary DNA (cDNA). The DNA was purified between enzymatic reactions and the size selection of the library was performed with AMPure XT beads (Beckman Coulter Genomics, Danvers, MA).

Libraries were assessed for concentration and fragment size using the DNA High Sensitivity Assay on the LabChip GX (Perkin Elmer, Waltham, MA). The library concentrations were also assessed by qPCR using the KAPA Library Quantification Kit (Complete, Universal) (Kapa Biosystems, Woburn, MA). The libraries were pooled and sequenced on a 100PE Illumina HiSequation 2500 run (Illumina).

### Alignment reads and coverage analysis

The reads obtained from the sequencing platforms were fed into the TopHat read alignment tool to be aligned to the *D. melanogaster* genomic reference sequence for each of the sequencing datasets. The reference genomic sequences were downloaded from the Ensembl project website (useast.ensembl.org). The TopHat alignment tool developed at the University of Maryland Center for Bioinformatics and Computational Biology was used to align the raw sequencing reads. TopHat v1.4 is a fast splice junction mapper for RNAseq reads (Trapnell *et al.* 2009). It aligns RNAseq reads to the reference genome using the ultrahigh-throughput short read aligner Bowtie, and then analyzes the mapping results to identify splice junctions between exons. The output from TopHat was obtained as BAM format files that consist of information on where the individual reads align within the reference genome and the splicing information of that read. In the alignment phase, we allowed up to two mismatches per 25 bp segment and removed reads that aligned to >20 genomic locations.

### Differential gene expression analysis

The TopHat alignments were then used to generate read counts for each gene in the reference genome annotation using HTSeq (Anders *et al.* 2015). The counts generated by HTSeq were subsequently used to generate the differential expression results using the R package DESeq.

### Transcript analysis using Cufflinks

Transcript abundances and splice variant identification for each sample was done using Cufflinks version 1.3 using the BAM alignment files obtained from TopHat (Ghosh and Chan 2016).

### Differential transcript analysis using CuffDiff

The BAM files from TopHat and the gtf files generated by cufflinks were used to identify differentially-expressed transcripts using CuffDiff (Trapnell *et al.* 2012). The results were filtered by FDR of <0.05, a FPKM value of >10, and a fold change of ±2.

### Gene Ontology (GO)

The Database for Annotation, Visualization and Integrated Discovery (DAVID) (Huang *et al.* 2009a,b) web service was used for GO analysis using the list of differentially-expressed genes. The p-value cut-off to determine enriched pathways was 0.1.

### Quantitative real-time RT-PCR (qRT-PCR) validation of genes

To validate differentially-expressed genes, we selected seven candidate genes based on significant fold differences and analyzed their mRNA levels using qRT-PCR. Four larvae from each treatment were collected at 6 and 24 hr after infection, and total RNA was extracted using the PrepEase RNA spin kit (Affymetrix) following the manufacturer’s instructions. RNA concentration was measured using a Nanodrop (Thermo Scientific) and samples were normalized to 350 µg. cDNA was synthesized using the High Capacity cDNA reverse transcription kit (Applied Biosystems) on a C1000 Thermal Cycler (Bio-Rad). cDNA samples were diluted 1:10 in nuclease-free water and 1 µl was used as a template for qRT-PCR experiments using the SsoAdvanced Universal *SYBR* *Green* Supermix (Bio-Rad). All experiments were carried out on a CFX96 Real-Time System (Bio-Rad). Primers (Table 1) for individual genes were designed using primer blast (NCBI) and annealing temperatures for each primer pair were estimated using a gradient PCR. All primers produced a single amplicon, and this was confirmed by both melting curve analysis and by visualizing the PCR product on the gel. Samples were run as technical duplicates and a total of three biological replicates were used for each treatment and time point. The cycling conditions included 95° for 2 min, 40 cycles of 95° for 15 sec, and an annealing step for 30 sec. The melting curve analysis consisted of an initial denaturation step at 95° for 15 sec, followed by an incremental temperature gradient from 65 to 95° for 15 sec at each temperature, with a ramp of 20 min from the lowest to the highest temperature. For each sample, the amount of mRNA detected was normalized to mRNA values of the housekeeping gene *RpL32*. The relative level of a given gene is represented as a ratio of 2^^CT(RpL32)^/2^^CT(Gene)^.

**Table 1:** List of primers used for qRT-PCR

Gene Name	Accession Number	Comments	Sequences
*TotC*	CG31508	Stress-induced humoral factor	Forward 5′-ACGTTGTCCCCTGAACAAAGG-3′
			Reverse 5′-TCCGACGTACTTGGTCTTTCG-3′
Unknown	CG31698	Unknown function	Forward 5′-CCAAACTTCCACCTCGGGAT-3′
			Reverse 5′-GATTCACGGGTTTGCTGTCG-3′
*IM3*	CG16844	Immune-induced molecule	Forward 5′-TTGGGTCTGCTGGCTCTG-3′
			Reverse 5′-TTCAACTGGCATCCTTCATTC-3′
Unknown	CG7248	Unknown function, contains a chitin-binding domain	Forward 5′-CAACACCTTCACCCACAGAAT-3′
			Reverse 5′-TTCACGCACAAGTAGAACTCATT-3′
*ImpE2*	CG1934	Unknown function	Forward 5′-AAGCCCGTTGCCTTGATCC-3′
			Reverse 5′-CTACTGGTGGCTCCTTATCCT-3′
*Sgs5*	CG7596	Unknown function	Forward 5′-TCAGAGCCTGAAATTGAATCCG-3′
			Reverse 5′-AAGAGCCCATTGGTAGTTCCT-3′
Unknown	CG3906	Unknown function, insect allergen-related	Forward 5′-AGCCACATTACATTGAGGTGTC-3′
			Reverse 5′-CGTGATCGGTTCTATTCGGATTG-3′
*RpL32*	CG7939	Ribosomal protein L32	Forward 5′-GATGACCATCCGCCCAGCA-3′
			Reverse 5′-CGGACCGACAGCTGCTTGGC-3′

### Statistical analysis

Results from qRT-PCR tests are represented as means and SDs of relative values from three biological replicates. Data were statistically analyzed using a one-way ANOVA with a Tukey *post hoc* test for multiple comparisons (GraphPad Prism 7).

### Data availability

The authors state that all data necessary for confirming the conclusions presented in the article are represented fully within the article.

## Results

### S. carpocapsae nematodes induce a large number of genes in D. melanogaster larvae

**Figure 1:**
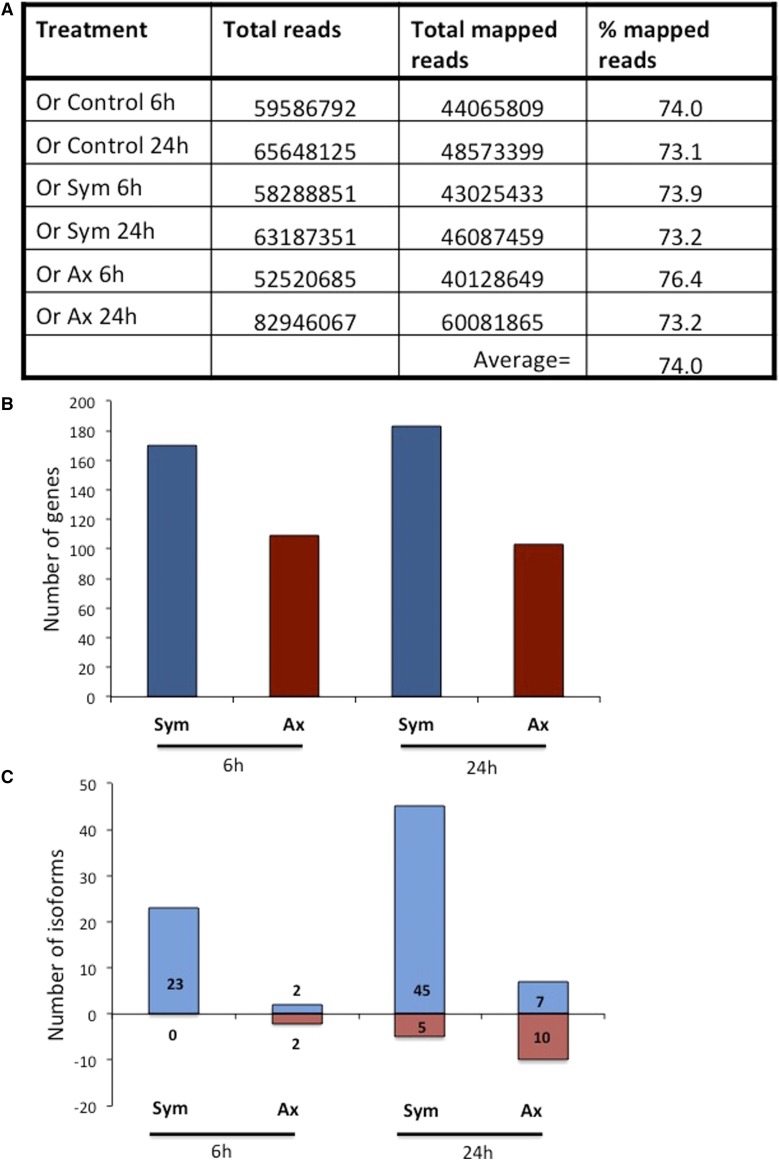
Infection of wild-type [Oregon (Or)] *D. melanogaster* third instar larvae with *S. carpocapsae* symbiotic (Sym) or axenic (Ax) nematodes induces a large number of transcripts. (A) Transcriptome summary (total number of reads, total number of mapped reads, and percentage reads mapped to the *D. melanogaster* genome) from larvae infected with *S. carpocapsae* symbiotic or axenic nematodes at 6 and 24 hr postinfection. (B) Number of differentially-expressed transcripts from larvae infected by *S. carpocapsae* symbiotic or axenic nematodes at 6 and 24 hr postinfection. (C) Cufflinks analysis of the differentially-expressed isoforms in *D. melanogaster* larvae infected with *S. carpocapsae* symbiotic or axenic nematodes at 6 and 24 hr postinfection.

We infected *D. melanogaster* wild-type Oregon larvae with 100 *S. carpocapsae* symbiotic or axenic IJs and generated the transcriptomic profile of larvae infected at an early (6 hr) and a late (24 hr) time point. Gene induction in infected larvae was relative to gene expression levels in uninfected larvae. The number of sequence reads mapped to an average of 74% of the *D. melanogaster* genome (Figure 1A).

We found that at 6 hr, symbiotic nematodes induced the expression of 170 genes and axenic nematodes induced 109 genes. We also found that at 24 hr, the number of genes induced by symbiotic nematodes increased slightly to 183 and those induced by axenic nematodes decreased to 103 (Figure 1B).

We also found that a large number of isoforms was differentially regulated upon infection with symbiotic nematodes compared to axenic nematodes. Symbiotic nematode infections upregulated 23 isoforms at 6 hr and 45 isoforms at 24 hr, while zero and five isoforms only were downregulated at 6 and 24 hr, respectively. In contrast, the number of isoforms induced by axenic nematodes was substantially lower. We found that infection with axenic nematodes upregulated two isoforms only at 6 hr and seven isoforms at 24 hr, and downregulated two and 10 isoforms at 24 hr, respectively (Figure 1C).

### S. carpocapsae nematodes modulate the induction of similar or different genes in D. melanogaster

**Figure 2:**
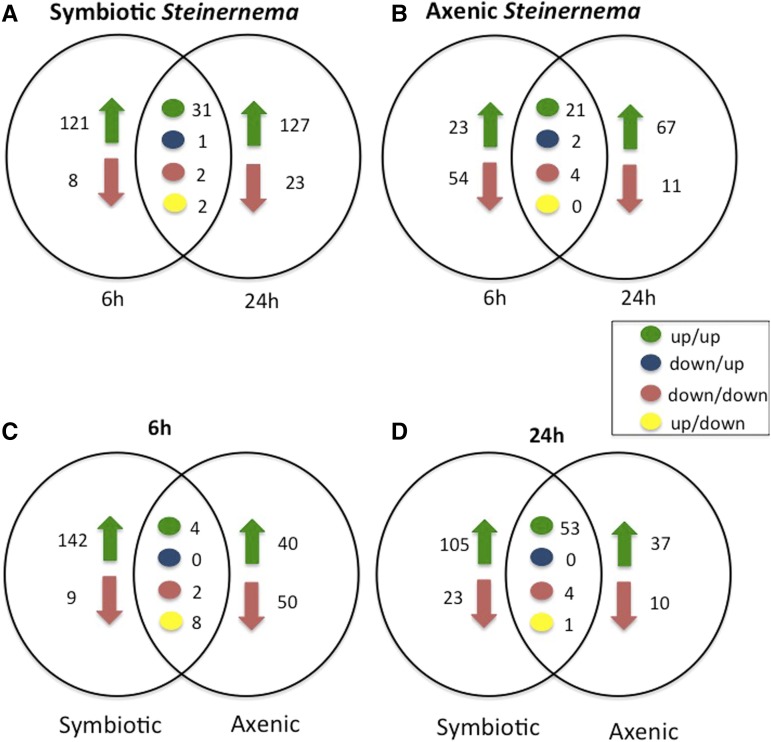
Infection with *S. carpocapsae* symbiotic or axenic nematodes induces distinct and shared transcriptomic profiles in *D. melanogaster* larvae. Venn diagrams showing the number of *D. melanogaster* differentially-expressed genes upon infection with *S. carpocapsae* (A) symbiotic nematodes at 6 and 24 hr, (B) axenic nematodes at 6 and 24 hr, (C) symbiotic and axenic nematodes at 6 hr, and (D) symbiotic and axenic nematodes at 24 hr. Expression patterns are indicated (up/up, gene upregulation at both 6 and 24 hr; up/down, gene upregulation at 6 hr and downregulation at 24 hr; down/up, gene downregulation at 6 hr and upregulation at 24 hr; and down/down, gene downregulation at both 6 and 24 hr).

We first investigated the number of common and distinct genes that are differentially regulated in *D. melanogaster* larvae upon infection with *S. carpocapsae* nematodes carrying or lacking *X. nematophila* bacteria. Upon infection with symbiotic nematodes, we found 121 and 127 upregulated genes at 6 and 24 hr, respectively. We also found eight and 23 downregulated genes at 6 and 24 hr postinfection, respectively. Interestingly, between the two time points, there were 36 shared genes, 31 of which were upregulated at 6 and 24 hr, one gene (*CG2229*) was downregulated at 6 hr and upregulated at 24 hr, two genes (*CR32658* and *CG31091*) were downregulated at both time points, and two genes (*CG42500* and *CG3763*) were upregulated at 6 hr but downregulated at 24 hr (Figure 2A). We also found that at 6 hr postinfection with axenic nematodes, 23 genes were upregulated and 54 were downregulated at 6 hr compared to 67 upregulated and 11 downregulated genes at 24 hr. Also, among the commonly regulated genes by axenic nematodes, 21 genes were upregulated and only two genes (*CG9070* and *CG44956*) were downregulated at 6 hr and upregulated at 24 hr, whereas four genes (*CG2559*, *CG6806*, *CG3292*, and *CR32658*) were downregulated at each time point (Figure 2B).

To identify changes in the number and types of genes that are differentially regulated by symbiotic and axenic nematodes, we compared the *D. melanogaster* genes that are induced early and late upon infection by the two types of nematodes. Interestingly, at 6 hr we found 142 upregulated and 9 downregulated genes in symbiotic nematode infections *vs.* 40 upregulated and 50 downregulated genes in axenic nematode infections. Also, of the common genes at 6 hr, four genes (*CG18444*, *CG16772*, *CG16844*, and *CG33337*) were upregulated, two genes (*CG32658* and *CG32071*) were downregulated by both symbiotic and axenic nematodes, and eight genes (*CG11650*, *CG3440*, *CG8502*, *CG7342*, *CG11089*, *CG7592*, *CG10078*, and *CG42500*) were upregulated by symbiotic nematodes and downregulated by axenic nematodes only (Figure 2C). We then compared the number of induced genes between symbiotic and axenic nematode infections at 24 hr and found that, although the number of upregulated genes was lower than those at the 6 hr time point, the number of commonly regulated genes between the two types of nematode infections was higher at 24 hr. Symbiotic nematode infections upregulated 105 genes and downregulated 23 genes. In contrast, 37 genes were upregulated and 10 genes (such as *CG3292*, *CG2736*, and *CG8745*) were downregulated upon axenic nematode infections. Among the commonly regulated genes at 24 hr, both types of nematodes upregulated 53 genes, four genes (*CG2559*, *CG4181*, *CG10513*, and *CR32658*) were downregulated by either axenic or symbiotic nematodes, and only one gene (*CG6271*) was upregulated by symbiotic nematodes and downregulated by axenic nematodes (Figure 2D).

These results indicate that *S. carpocapsae* axenic and symbiotic nematodes regulate a large variety of similar or distinct types of genes at early and late times postinfection of *D. melanogaster* larvae.

### S. carpocapsae infection regulates several molecular pathways and biological activities in D. melanogaster

**Figure 3:**
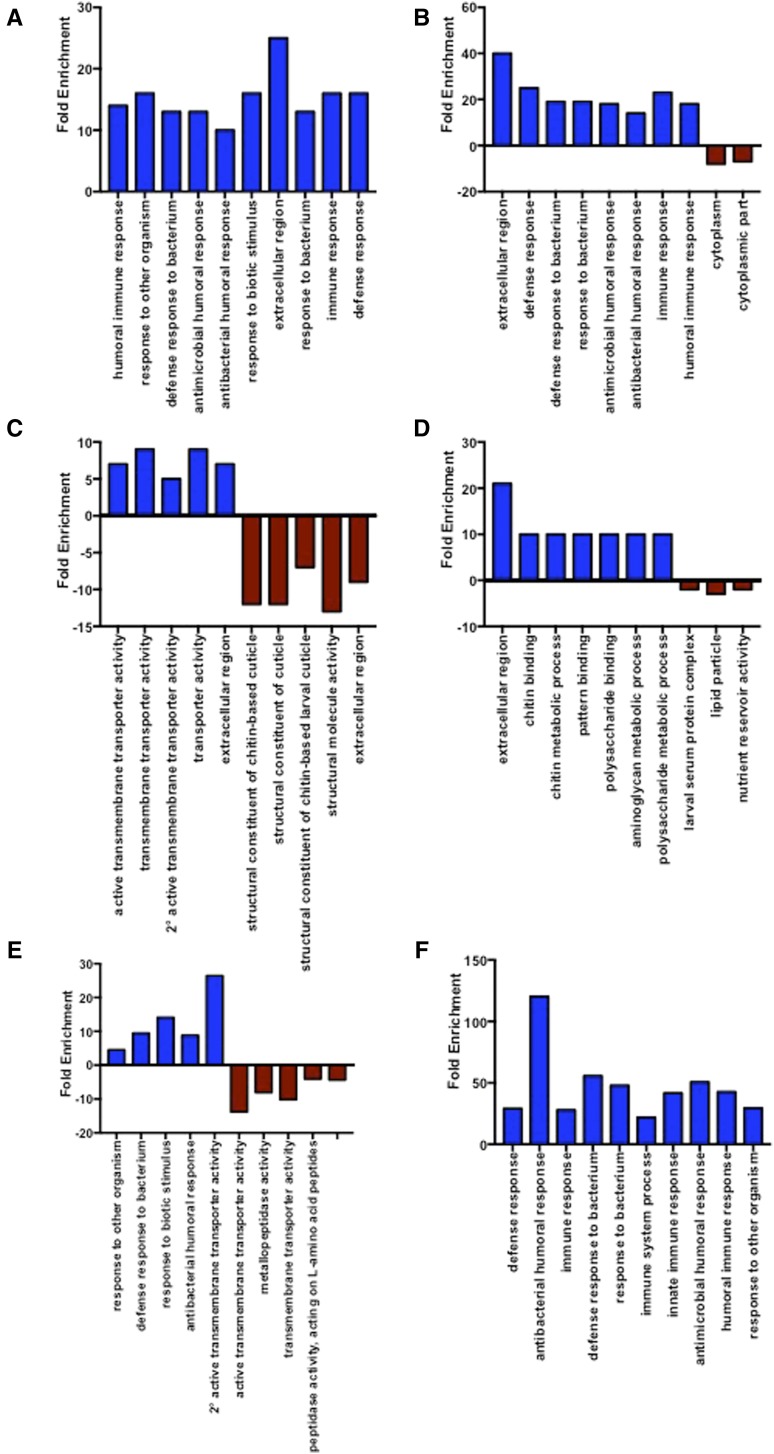
Infection with *S. carpocapsae* symbiotic (Sym) or axenic (Ax) nematodes induces diverse physiological responses and biological activities in *D. melanogaster* larvae. This is characterized by the enrichment of pathway-specific genes based on their molecular and biological functions using the DAVID (Database for Annotation, Visualization and Integrated Discovery) classification database. Representative categorization of genes in larvae infected with *S. carpocapsae* (A) symbiotic nematodes at 6 hr, (B) symbiotic nematodes at 24 hr, (C) axenic nematodes at 6 hr, (D) axenic nematodes at 24 hr, and comparison of axenic and symbiotic infections at (E) 6 hr and (F) 24 hr postinfection.

We conducted the GO analysis using the DAVID database to identify the molecular pathways and biological activities that are involved in the *D. melanogaster* larval response to infection by *S. carpocapsae* nematodes (Figure 3). We found that infection of *D. melanogaster* larvae with symbiotic or axenic nematodes elicited the enrichment of several specific and overlapping categories of genes at each time point postinfection. For example, at 6 hr we found that infection with symbiotic nematodes induced the enrichment of genes involved in the humoral immune response and serine-type endopeptidase activity (Figure 3A and Supplemental Material, Figure S1A), whereas infection with axenic nematodes upregulated genes with transmembrane transporter activity and downregulated genes in the chitin-based cuticle pathway (Figure 3B and Figure S1B).

We also found that infection with symbiotic nematodes at 24 hr upregulated genes related to the immune system process, polysaccharide binding, aminoglycan metabolic process, and cell wall macromolecule catabolic process, and downregulated genes associated with the carbohydrate metabolic process and apoptosis signaling (Figure 3C and Figure S1C). Also, infection with axenic nematodes at 24 hr upregulated genes in similar pathways as well as genes related to neuroactive ligand–receptor interaction. The downregulated genes mainly belonged to larval serum protein complex, lipid particle, and nutrient reservoir activity (Figure 3D and Figure S1D).

We then compared the enrichment of genes between symbiotic and axenic nematode infections at 6 and 24 hr time points. At 6 hr postinfection, we observed that a large number of upregulated genes belonged to immune defense responses and that the downregulated genes encoded metalloproteases and hydrolases (Figure 3E and Figure S1E). Conversely, at 24 hr, we found that all gene categories were enriched and with the exception of lipid metabolic processes, the rest of the enriched genes belonged to a variety of immune response categories, such as the immune system process and antimicrobial humoral response (Figure 3F and Figure S1F).

Thus, the pathway analysis revealed that infection with symbiotic nematodes enriched genes related to immune functions in *D. melanogaster*, whereas infection with axenic nematodes induced the enrichment of genes belonging to a variety of categories ranging from polysaccharide binding to the chitin metabolic process. These results provide novel insights into the molecular processes that take place in *D. melanogaster* larvae upon infection by entomopathogenic nematodes carrying or lacking their associated bacteria and contribute toward a better understanding of the molecular events that take place in the host during nematode infection.

### S. carpocapsae nematodes affect key immune and developmental processes in D. melanogaster larvae

**Figure 4:**
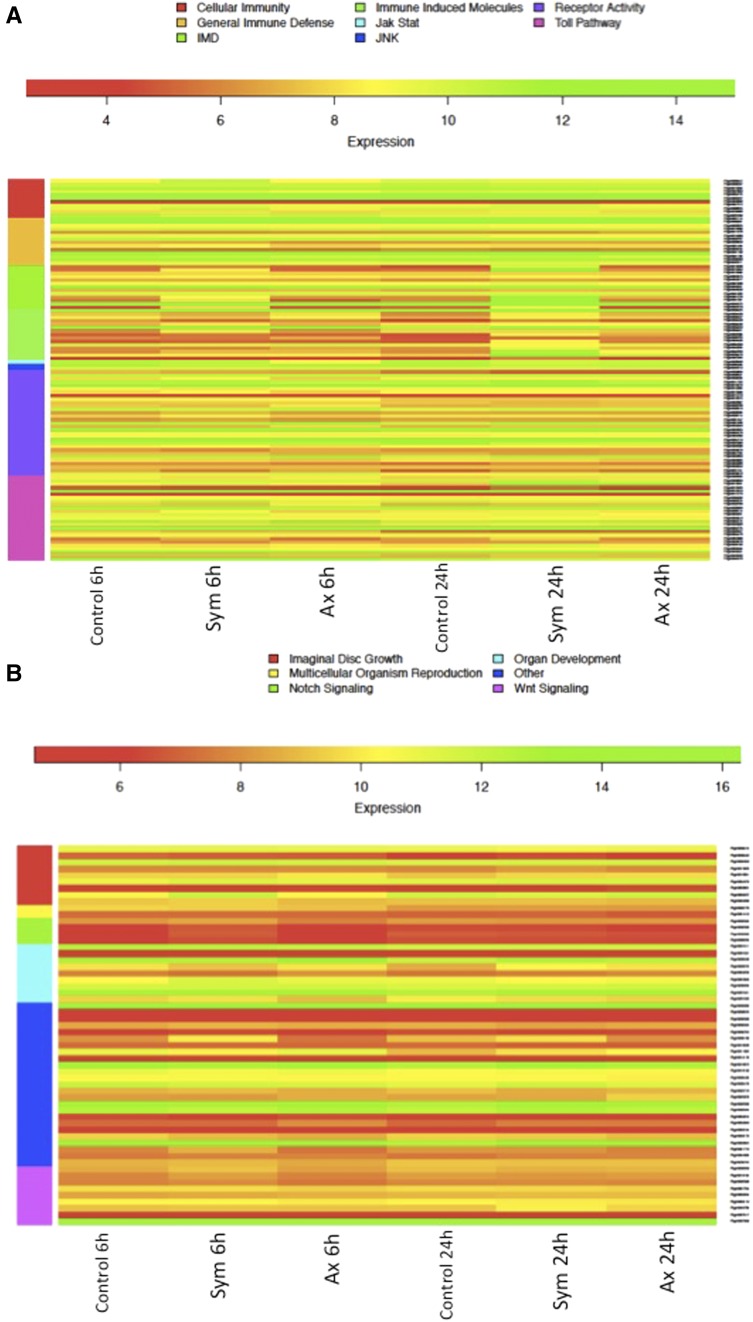
Infection with *S. carpocapsae* symbiotic or axenic nematodes differentially regulates the transcription of a variety of immune and developmental genes in *D. melanogaster* larvae. Genes selected from the Gene Ontology (GO) analysis have a positive expression level as an indication of their upregulation upon infection with the nematode parasites. The selected genes are categorized into: (A) Immune Genes, and they are further grouped into genes with cellular immune functions, genes encoding immune-induced molecules, genes with receptor activity, or genes that are regulated by the immune deficiency (IMD), Toll, Janus Kinase and Signal Transducer and Activator of Transcription (JAK/STAT), or cJun-N-terminal Kinase (JNK) pathways; and (B) Developmental Genes, and they are further grouped into genes that have functions in multicellular organism reproduction and organ development, genes that are regulated by the Notch or Wnt signaling pathways, genes that belong to the Imaginal Disc Growth Factors family, or genes with other functions in development.

To identify what immune and developmental genes are regulated upon infection by *S. carpocapsae* nematodes, we generated a heat map to illustrate the differential gene transcription levels for both types of nematode infections and time points (Figure 4, A and B). For the heat map with the immunity-related genes, we included genes from the four known immune signaling pathways [Immune Deficiency (IMD), Janus Kinase and Signal Transducer and Activator of Transcription (JAK/STAT), cJun- N-terminal Kinase (JNK), and Toll], genes involved in cellular immune responses and hematopoiesis, immune-induced molecules, genes with immune receptor activity, and genes with general immune functions, which also included genes with putative immune roles (Figure 4A).

In the Toll pathway (Valanne *et al.* 2011), the Gram-negative bacteria binding protein 3 (GNBP3) was downregulated in larvae infected by axenic nematodes at 6 hr compared to the uninfected control larvae, and it was upregulated at 24 hr postinfection in larvae infected by either symbiotic or axenic nematodes. We found that at 6 hr, the Toll pathway protein *Serpin*-27A was upregulated by symbiotic nematodes and downregulated by axenic nematodes. Conversely, this serpin gene was upregulated at 24 hr by either symbiotic or axenic nematodes. The Toll immune-regulated protein Fondue was downregulated by both types of nematodes at 6 hr, but its expression increased at 24 hr postinfection. The AMP-coding gene *Drosomycin* (Zhang and Zhu 2009) was upregulated at both 6 and 24 hr in larvae infected by symbiotic nematodes, but it was downregulated at 6 hr and upregulated at 24 hr by axenic nematodes.

In the IMD pathway (Kaneko *et al.* 2005), certain recognition protein genes were induced at different levels by either symbiotic or axenic *S. carpocapsae*. We found that PGRP-SC1a/b (Garver *et al.* 2006) was slightly downregulated at 6 hr in larvae responding to symbiotic or axenic nematodes, it was upregulated at 24 hr in response to axenic nematodes, and showed no changes in expression with symbiotic nematodes. In contrast, *PGRP-SC2* (Bischoff *et al.* 2006) and *PGRP-LB* (Zaidman-Rémy *et al.* 2006) were strongly induced by both symbiotic and axenic nematodes at 24 hr postinfection, but showed little to no change at the 6 hr time point. We also found that *kenny* (Silverman *et al.* 2000) and the IMD pathway transcription factor *Relish* (Stöven *et al.* 2000) were highly induced in larvae infected for 24 hr by either type of nematode. The IMD-controlled AMPs Attacin-A, Attacin-B, and Attacin-C were strongly upregulated by symbiotic nematodes at both 6 and 24 hr, and only slightly upregulated at 24 hr by axenic nematodes.

We also found that JAK/STAT and JNK pathway genes were not induced as strongly as genes in the Toll and IMD signaling pathways. We found that *Turandot-A* (*Tot-A*) (Ekengren and Hultmark 2001) expression was increased by symbiotic nematodes at 6 hr and decreased at 24 hr compared to the uninfected control. However, axenic nematode infections caused the downregulation of *Tot-A* at 6 hr and its upregulation at 24 hr. The JNK pathway gene *puckered* (McEwen and Peifer 2005) was significantly upregulated at 24 hr in axenic nematode infected larvae, but it was only slightly upregulated by symbiotic nematodes. Interestingly, the expression of genes with receptor activity, such as *peste* (Nakanishi and Shiratsuchi 2006), *scavenger receptor class V*, *type 1*, and *Toll 5* (Tauszig *et al.* 2000), were highly increased by axenic nematodes at 24 hr and at lower levels by symbiotic nematodes. In contrast, the expression of genes such as *Lapsyn*, *Gp150*, *diuretic hormone 31*, and *CG5096* was significantly increased only by axenic nematode infections at 24 hr. We also found that genes involved in cellular immune responses were upregulated by symbiotic and axenic nematodes. The zinc finger protein *jing* and *singed* were strongly induced by symbiotic nematodes at 6 hr, whereas *serine protease 7* was strongly induced at 24 hr. We further noticed that other genes such as *nitric oxide synthase* and *ATP-dependent RNA helicase p62* were upregulated by axenic nematodes at 24 hr, whereas *hemese* (Kurucz *et al.* 2003) was upregulated at both 6 and 24 hr. Interestingly, *Tep1* and *Tep2* (Bou Aoun *et al.* 2010) were strongly induced by symbiotic nematodes at both 6 and 24 hr.

To identify the *D. melanogaster* developmental genes that are differentially-regulated due to *S. carpocapsae* infection, we generated a heat map to illustrate the differential gene expression levels in larvae infected by symbiotic or axenic nematodes at each time point postinfection (Figure 4B). We included genes belonging to the *Idgf* family, multicellular organism development, organ development, Notch, and Wnt signaling pathways. In the Idgf category, axenic nematodes strongly induced the expression of *lamina ancestor* and *bursicon* genes at 24 hr, and *tenectin* at 6 hr. The gene *lethal (3) malignant blood neoplasm* was upregulated by both symbiotic and axenic nematodes at 24 hr, and *E(spl) region transcript m2* of Notch signaling was upregulated by symbiotic nematodes at 6 hr. We also found differential expression of genes regulating organ development functions. *Lonely heart* and *LDLa domain containing chitin binding protein-1* was induced by axenic nematodes at 24 hr and *matrix metalloproteinase* 1 was induced by symbiotic nematodes at 6 hr. Interestingly, *pericardin* and *scab* were strongly induced by both axenic and symbiotic nematodes at 24 hr; however, induction levels by axenic nematodes were higher compared to those by symbiotic nematodes, with the exception of *CG17278* (Wnt signaling), which was highly induced by symbiotic nematodes at 24 hr. We also estimated the expression of other developmental genes and found that the gene *punch* was upregulated by symbiotic nematodes at 6 hr and downregulated by axenic nematodes at 24 hr. *Yellow-F* and *glial cells missing* was induced only by symbiotic nematodes at 6 hr, and the tissue inhibitor of *metalloproteases* was induced by axenic nematodes at 24 hr. Several other genes were induced by both symbiotic and axenic nematodes at 24 hr. For example, *viking*, *ejaculatory bulb III*, *collagen type IV*, and *SPARC* were all induced at higher levels by axenic nematodes, while symbiotic nematodes were responsible for the stronger induction of *CG7714*. We used specific primers for seven genes (Table 1) and validated the RNAseq results using qRT-PCR (Figure S2).

### S. carpocapsae infection affects D. melanogaster genes conserved in M. sexta and humans

**Figure 5:**
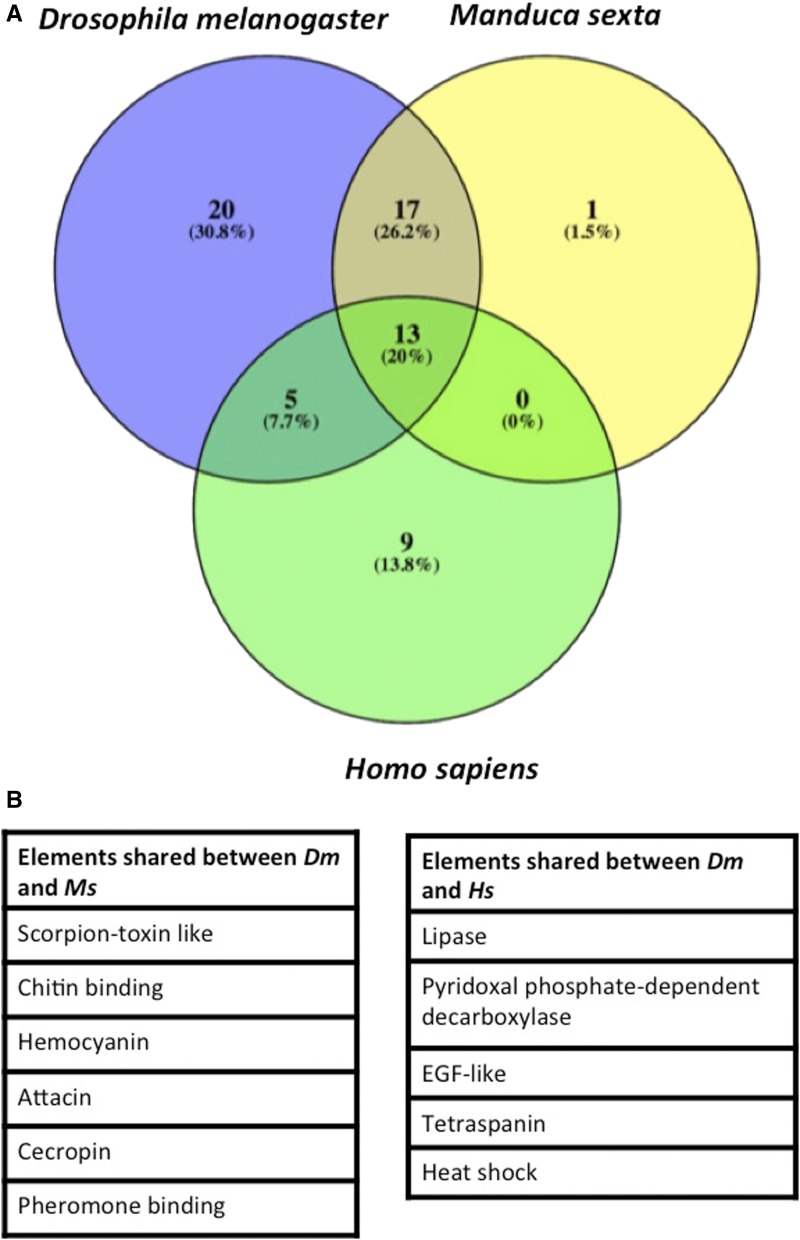
(A) Orthologs of the top 55 differentially transcribed *Drosophila melanogaster* genes (*Dm*) in *Manduca sexta* (*Ms*) and *Homo sapiens* (*Hs*). *D. melanogaster* genes were selected from the Gene Ontology (GO) analysis that were either up- or downregulated upon infection by *S. carpocapsae* axenic or symbiotic nematodes, and the protein domains were selected based on the UniProt IDs (identifiers). (B) Table showing the protein domains shared between *Dm* and *Ms*, and those that are shared between *Dm* and *Hs*. EGF, endothelial growth factor.

To investigate whether the *D. melanogaster* genes induced by *S. carpocapsae* infection have known functions in other organisms, we selected the top 55 most differentially-expressed genes in nematode-infected larvae (Figure 5). We included hits based on the UniProt database for a natural host of entomopathogenic nematodes, the tobacco hornworm *Manduca sexta*, and human (*Homo sapiens*). We used Venn diagrams to depict the conservation of genes based on shared or distinct protein domains in these three organisms.

We observed that of the 55 *D. melanogaster* protein-coding genes, 20 were exclusive to *D. melanogaster*, one to *M. sexta*, and nine to *H. sapiens*. We also found that 17 of these 55 protein-coding genes in *D. melanogaster* also exist in *M. sexta* and they belong to proteins with scorpion toxin-like activity, chitin binding properties, hemocyanin, attacin, and pheromone-binding domains. However, the five domains shared between *D. melanogaster* and *H. sapiens* belong to lipase, pyridoxal phosphate-dependent decarboxylase, EGF-like, tetraspanin, and heat shock proteins. Interestingly, the 13 common elements between all three organisms possess certain domains, such as amidase, kazal, serpin, or kunitz.

The results from this analysis show that certain *D. melanogaster* genes induced upon infection with *S. carpocapsae* nematodes have orthologs in two other organisms, *M. sexta* (insect host) and *H. sapiens* (humans), indicating conservation in their potential roles against parasitic nematodes.

## Discussion

Here, we present the transcriptional profile of *D. melanogaster* larvae infected by the potent nematode parasite *S. carpocapsae* containing or lacking its mutualistic *X. nematophila* bacteria. We report the identification of several types of *D. melanogaster* genes that are differentially regulated in the larval stage during interaction with either type of nematode. Initial characterization of the *D. melanogaster* transcriptome reveals that the number and nature of genes induced upon infection by axenic or symbiotic nematodes is substantially different. This suggests that *S. carpocapsae* nematodes, in the absence or presence of their associated bacteria, elicit different types of immune reactions because they employ distinct strategies to infect insects and interfere with their immune system. We have also identified specific genes that are significantly up- or downregulated during nematode infection. We have found that these genes have conserved functions in the natural host of the entomopathogenic nematodes *S. carpocapsae*, larvae of the lepidopteran *M. sexta*, as well as in humans, and therefore might possess conserved antinematode properties.

A recent transcriptome analysis on *D. melanogaster* adult flies infected by *H. bacteriophora* symbiotic or axenic nematodes, or their associated *P. luminescens* bacteria alone, has identified a wide variety of genes that are differentially regulated in response to the pathogens. These genes are mainly related to the stress response, lipid homeostasis, metabolic processes, and neuronal functions (Castillo *et al.* 2015). Interestingly, some of these genes were reported to form factors with potential roles in host antinematode and antibacterial immune responses. Also, a previous transcriptome study on *D. melanogaster* larvae infected by symbiotic *H. bacteriophora* nematodes only showed that genes encoding complement factors as well as recognition and extracellular matrix proteins were expressed at high levels (Arefin *et al.* 2014). Here, we included infections of *D. melanogaster* larvae with axenic *S. carpocapsae* nematodes to identify the *D. melanogaster* genes that are differentially regulated in response to the nematodes without the input of their mutualistic *X. nematophila* bacteria. We have found that most *D. melanogaster* genes and isoforms are differentially regulated in response to symbiotic nematodes compared to axenic worms, suggesting the additional contribution of mutualistic *X. nematophila* in the interaction with the insect immune system during infection with the nematode–bacteria complexes.

Our analysis shows that a subset of *D. melanogaster* induced genes is common between the two types of nematode infections, compared to a larger number of genes that are distinct either to axenic or symbiotic *S. carpocapsae* infection. In addition, early in the infection process, axenic nematodes downregulate a larger number of genes compared to those downregulated by symbiotic nematodes, but as the infection progresses the number of downregulated genes increases in larvae infected by symbiotic worms. This suggests that the insect immune system can be compromised by entomopathogenic nematodes devoid of their associated bacteria, especially during the initial stages of infection.

Upon infection with axenic or symbiotic *S. carpocapsae*, we found strong induction of several Heat Shock Protein (*hsp*)-coding genes, which can be attributed to the insect response to stress conditions during nematode penetration, invasion, and migration in the insect, which is accompanied by severe tissue damage (Feder and Hofmann 1999; Sorensen *et al.* 2005). It was recently shown that *H. bacteriophora* nematodes use a specialized buccal protruding tooth to penetrate through the *D. melanogaster* larval cuticle and the gut epithelium, thus causing extensive wounding to those tissues (Ciche *et al.* 2008; Arefin *et al.* 2014). Based on our findings, certain *hsp* genes, such as *Hsp23* and *Hsp27*, are strongly upregulated by *S. carpocapsae* symbiotic nematodes and show little to no change in response to axenic nematodes at 6 hr postinfection. In contrast, at 24 hr postinfection, these genes are upregulated in response to axenic nematodes only. These results indicate that both the nematode–bacteria complexes, as well as the nematodes alone, are capable of causing physical damage to the larvae thereby leading to the strong induction of *hsp* genes. We further observed a strong induction of *TotC*, an immune and stress response gene of the *Turandot* family, upon infection by either symbiotic or axenic nematodes (Ekengren and Hultmark 2001). Similarly, *Hsp* and *TotC* were also previously detected in *D. melanogaster* adult flies upon infection by either symbiotic or axenic *H. bacteriophora* nematodes, as well as *P. luminescens* bacteria only (Castillo *et al.* 2015). These results confirm that entomopathogenic nematode infection in *D. melanogaster* adult flies as well as larvae leads to the potent induction of several stress factors.

Classifying the genes that are induced by either type of *S. carpocapsae* nematode reveals that symbiotic nematodes primarily induce genes with immune-related functions, whereas axenic nematodes induce genes encoding peptidases, as well as chitin-binding and structural components of the larval cuticle. A particular class of genes that are induced by axenic nematodes can be grouped into the category “structural constituent of the insect peritrophic membrane.” This membrane consists of chitin and peritrophin-like proteins that line the insect gut to modulate gut immune responses in the host against bacterial infections (Lehane 1999; Hedegus *et al.* 2009; Buchon *et al.* 2009; Kuraishi *et al.* 2011). The strong induction of genes that are mainly expressed in the peritrophic membrane (*peritrophin-15a* and *-15b*) upon axenic nematode infection suggests that they might be involved in the insect response against nematodes free from *X. nematophila* bacteria.

In the *D. melanogaster* gut, the Imd pathway is responsible for the induction of AMPs in response to microbial infections (Myllymäki *et al.* 2014). AMP induction is the result of the interaction of the PGRPs and the pathogen-specific PGN, thereby initiating the intracellular molecular cascade (Casanova-Torres and Goodrich-Blair 2013). Both PGRP-LB and PGRP-SC2 are among the five PGRPs that can process DAP-type PGN (Dziarski and Gupta 2006), and their induction suggests that *S. carpocapsae* axenic nematodes or certain molecules that they produce are recognized by these IMD pathway receptors, but apparently fails to induce certain AMP effectors or induces Attacin-A, -B, or -C at low levels. Conversely, these AMPs were strongly induced in response to symbiotic nematodes, suggesting that the detection of nematode–bacteria complexes was likely due to the identification of *X. nematophila* by the insect PGRPs.

Contrary to the number and induction level of genes related to humoral immune responses, we found very few genes with known function in cellular immune processes that were differentially regulated by *S. carpocapsae* nematode infection. A possible explanation for this result could be that the *D. melanogaster* cellular immune response is probably not crucial against infection by *S. carpocapsae*, or that the molecules secreted by these nematodes are effective in suppressing or preventing the hemocyte action that regulates insect cellular immune processes (Brivio *et al.* 2005). Interestingly, a previous study has shown that *G. mellonella* hemocytes were able to respond to infection by *H. bacteriophora* but not to *S. carpocapsae*, suggesting that these nematodes are able to evade the insect cellular immune response. The encapsulation response in insects is facilitated by the ability of hemocytes to spread and adhere to the nematode surface (Stanley *et al.* 2012). *S. carpocapsae* nematodes have been shown to produce certain proteases and other factors that impair clot formation, thereby evading the insect melanization response and eicosanoid biosynthesis (Stanley *et al.* 2012; Toubarro *et al.* 2013). Eicosanoids and their related lipids have been found to participate in the immune response of *D. melanogaster* larvae in response to infection by *H. bacteriophora* nematodes (Hyrsl *et al.* 2011). Here, we have observed induction of certain genes belonging to the melanization response in larvae infected by *S. carpocapsae* symbiotic or axenic nematodes. The induction of *ppo1*, *ppo2*, and *pro-PO A1* in response to both symbiotic and axenic nematodes is in contrast to the upregulation of genes such as *black cells* encoding prophenoloxidase (Gajewski *et al.* 2007) and *phenoloxidase subunit A3* in response to axenic nematodes only. Taken together, it can be argued that the wound healing and clotting responses in the *D. melanogaster* larvae upon infection by *S. carpocapsae* nematodes are probably not entirely dependent on the action of the *ppo* genes, and might involve the contributions of other genes that have not yet been identified or fully characterized.

Categorizing the strongly induced genes into those having immune or developmental-related function revealed the nature of genes from each category. We found that certain genes previously reported to function in developmental processes were highly induced in *D. melanogaster* larvae in response to infection by *S. carpocapsae* axenic or symbiotic nematodes. One of those genes was *pericardin* (*prc*), a mammalian collagen IV homolog (Chartier *et al.* 2002). Interestingly, at 24 hr postinfection, we found a strong upregulation of *prc* in response to symbiotic nematode infections and an even stronger induction in response to axenic nematodes. The function of *prc* in *D. melanogaster* organ development is to decorate the heart tube, regulate heart morphogenesis, and maintain cardiac integrity (Zaffran *et al.* 1995; Chartier *et al.* 2002). We also find strong induction of the gene *lonely heart* (*loh*), which is responsible for the recruitment of PRC to the extracellular matrices of different tissues in order to regulate the assembly of the matrices. A previous study has shown that the normal functioning of both *prc* and *loh* is crucial for the cellular behavior and proper functioning of the organs in *D. melanogaster* (Drechsler *et al.* 2013). We found that *loh* exhibits stronger induction levels in response to *S. carpocapsae* axenic nematodes compared to symbiotic nematodes at the late stage of infection. We also found strong induction of the collagen homologs Collagen type IV α 1 and Viking at a later time point postinfection by both symbiotic and axenic nematodes. It was recently shown that Viking, as well as the basement membrane protein glutactin, function together in would healing in the *D. melanogaster* larvae infected by *H. bacteriophora* nematodes (Arefin *et al.* 2014). Therefore, it can be argued that *prc* and *loh* might also be involved in wound healing or clotting responses in *D. melanogaster* larvae against infection by *S. carpocapsae* nematodes.

Although *S. carpocapsae* can naturally infect a wide range of insect species (Lacey *et al.* 2015), *D. melanogaster* has not yet been found to act as host to this nematode species. Previous studies have examined the transcriptional regulation of the immune response of certain *Drosophila* species to natural parasites and microbial pathogens. The results suggest that *Drosophila* adult flies and larvae can trigger different immune genes and pathways against natural viral pathogens (Habayeb *et al.* 2006; Carpenter *et al.* 2009; Kemp *et al.* 2013), bacterial pathogens (Vodovar *et al.* 2005), and endoparasitoid wasps (Wertheim *et al.* 2005). The *Drosophila* immune response can vary from activating peptidoglycan recognition proteins and antimicrobial peptides through NF-κB signaling pathways to reactions that are restricted against specific types of natural pathogens (Keebaugh and Schlenke 2014). Here, we have also shown that *S. carpocapsae* nematodes not only interfere with the expression of genes with known immune roles in *D. melanogaster*, but also with genes with unexplored function in the fly immune system. These findings imply that *D. melanogaster* has developed particular mechanisms to respond to *S. carpocapsae* nematodes, probably due to a lack of host–parasite coadaptation and coevolution.

In conclusion, we have shown that *S. carpocapsae* nematodes are able to trigger the *D. melanogaster* larval immune system even in the absence of their *X. nematophila* mutualistic bacteria. We have shown that *D. melanogaster* larvae activate several different types of genes in response to *S. carpocapsae* nematode infection. These include genes with known immune function, genes involved in developmental processes, as well as genes with unknown mechanistic roles, especially in the interaction of the insect immune system with entomopathogenic nematodes. Our transcriptome study has shed more light on the nature of insect genes that are induced in response to potent nematode parasites. Infection of *D. melanogaster* larvae by axenic or symbiotic *S. carpocapsae* nematodes has revealed the induction of unique genes that are not shared with other infection models. Similar transcriptome studies will lay the foundation for testing the candidate genes through functional studies that will promote our understanding of the molecules that modulate the interaction between insects and parasitic nematodes.

## Supplementary Material

Supplemental material is available online at www.g3journal.org/lookup/suppl/doi:10.1534/g3.117.041004/-/DC1.
