# Supplementary figures and images for: RNAseq Analysis of the Drosophila Response to the Entomopathogenic Nematode Steinernema
Source: G3 (Bethesda). 2017 Apr 24;7(6):1955–67. doi: 10.1534/g3.117.041004 (PMC5473771; doi:10.1534/g3.117.041004)

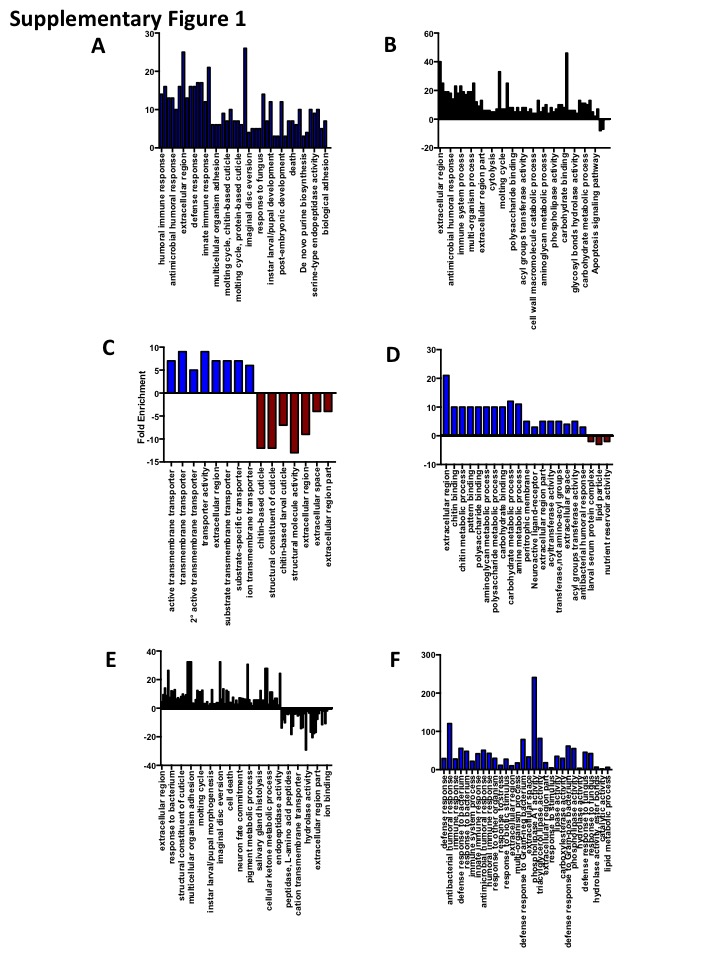

Supplement: Supplementary file 1 [file 1955FigureS1.jpg]

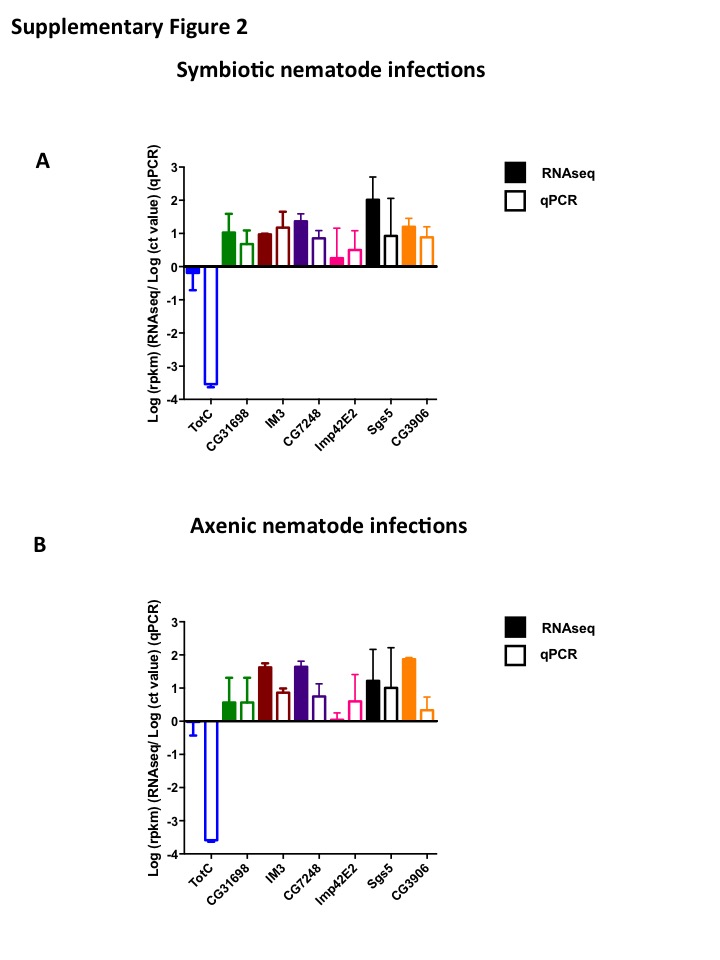

Supplement: Supplementary file 2 [file 1955FigureS2.jpg]
